# *In vitro* effect of immune regulatory cytokines on vitiligo pathogenesis

**DOI:** 10.1186/1471-2164-15-S2-P39

**Published:** 2014-04-02

**Authors:** Mala Singh, Mohmmad Shoab Mansuri, Naresh C Laddha, Mitesh Dwivedi, Yogesh S Marfatia, Rasheedunnisa Begum

**Affiliations:** 1 Department of Biochemistry, Faculty of Science, The Maharaja Sayajirao University of Baroda, Vadodara, Gujarat, India; 2 Department of Skin and V.D., Faculty of Medicine, The Maharaja Sayajirao University of Baroda, Vadodara, Gujarat, India

## Background

Vitiligo is an acquired, hypomelanotic skin disorder characterized by circumscribed de-pigmented macules resulting from the loss of functional melanocytes. Various factors which may be responsible for precipitating this disorder in susceptible patients are oxidative stress, auto-immunity and neurochemicals.

## Materials and methods

The skin samples were obtained with the consent of healthy individuals. Isolation of melanocytes was done according to the standard method [1] and normal human melanocytes (NHM) were grown in basal medium supplemented with growth factors. Dose dependent effect of different cytokines such as TNFα, IL6 and IL10 on NHM growth and proliferation was studied. MTT assay, RNA isolation, cDNA synthesis and relative gene expression studies were performed as described. This study was approved by the Institutional Ethical Committee for Human Research (IECHR), The M. S. University of Baroda, Vadodara, Gujarat, India.

## Results

**Figure 1:**
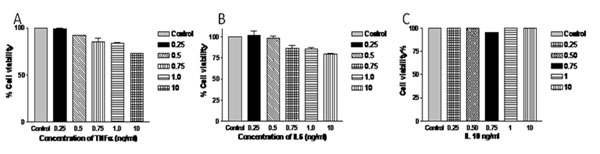
Dose dependent effect of TNFα (A), IL6 (B) and IL10 (C) on normal human melanocyte growth and proliferation.

**Figure 2:**
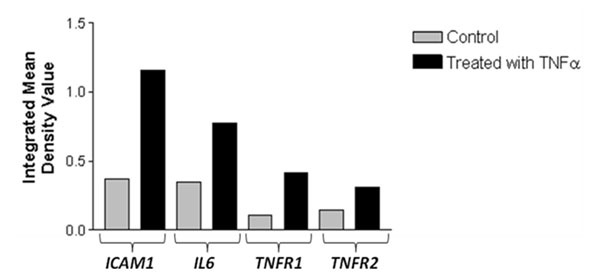
TNFα induced expression of *ICAM1*, *IL6*, *TNFR1* and *TNFR2.*

The pro-inflammatory cytokines (TNFα and IL6) induced 37% & 20% cell death respectively in NHM, on the other hand the anti-inflammatory cytokine, IL10 did not affect the growth of NHM (Fig.1). Our earlier studies have shown high systemic mRNA and protein levels of TNFα and TNFβ in Gujarat vitiligo patients compared to controls [2,3]. We have studied dose dependent effect of TNFα on NHM, and found that TNFα induced cell death in a dose dependent manner. Interestingly, higher concentrations of TNFα induced up-regulation of its receptors *TNFR1* &*TNFR2* along with significant increase in *IL6* and *ICAM1*expression (Fig. 2). IL6 was also found to increase the expression of *ICAM1*[4], which favors the attachment of T cells and melanocytes and thus making the latter more susceptible for auto-immune destruction. We have also found the synergistic effect of TNFα and IL6 in inducing NHM apoptosis. In addition, TNFα and IL6 were found to aggravate their effects under oxidative stress.

## Conclusions

The present study reveals that TNFα significantly induces *IL6*, *ICAM1*, *TNFR1* and *TNFR2* expression. In addition IL6 also induces *ICAM1* expression [4]. ICAM1 enhances T-cell and melanocyte attachment, thus augmenting melanocyte destruction by immune system. Under oxidative stress, which mimics the microenvironment of vitiligo, *TNFA* is found to enhance apoptosis of melanocytes which would result in de-pigmentation of the skin. Thus, our *in vitro* studies further strengthen the scientific evidences linking oxidative stress and immune system to vitiligo pathogenesis giving credence to a convergent terminal pathway of oxidative stress-autoimmunity mediated melanocyte loss.
